# Syndrome of inappropriate antidiuretic hormone secretion associated with prolonged keterolac use 

**DOI:** 10.5414/CNCS108083

**Published:** 2014-01-22

**Authors:** Sian Yik Lim, Ragesh Panikkath, Sharma Prabhakar

**Affiliations:** Department of Internal Medicine, Texas Tech University Health Sciences Center, Lubbock, TX, USA

**Keywords:** keterolac, nonsteroidal anti-inflammatory drugs, syndrome of inappropriate antidiuretic hormone secretion, prostaglandins

## Abstract

Nonsteroidal anti-inflammatory drugs (NSAIDs) are commonly used analgesics. Although rare, clinicians need to keep in mind that their use may precipitate hyponatremia and syndrome of inappropriate antidiuretic hormone (SIADH), especially in high-risk patients with multiple comorbidities. In the kidneys, prostaglandins attenuate the water retention effect of antidiuretic hormone. NSAIDs cause a decrease in prostaglandins in the kidney and therefore the effect of ADH is potentiated. We report a case of SIADH that was associated with keterolac in a 65-year-old male. SIADH has not previously been reported with keterolac, a strong NSAID with comparable analgesic effect as morphine and meperidine. Keterolac may have unique properties different from other NSAIDS which may predispose to the development of hyponatremia. In our case, prolonged use of keterolac may have contributed to the development of SIADH and caution is needed when keterolac is used for prolonged duration. A review of the literature regarding development of SIADH and hyponatremia in the setting of NSAIDs is also presented.

## Introduction 

Nonsteroidal anti-inflammatory drugs (NSAIDs) are being increasingly used in the general population in the United States [1]. The major side effects of NSAIDs include gastrointestinal toxicity, acute renal failure, hypertension, and peripheral edema [2]. Syndrome of inappropriate antidiuretic hormone (SIADH) and hyponatremia associated with NSAID is a rare occurrence, but clinicians need to be aware of this possibility especially when evaluating cases of hyponatremia. We report a case of SIADH associated with keterolac, a NSAID with strong analgesic effects. Although hyponatremia and SIADH have been reported with other NSAIDs [3], we are not aware of any previous reports of SIADH associated with keterolac. 

## Case 

Our patient is a 65-year-old male with past medical history of chronic obstructive pulmonary disease (COPD), hypertension, and hyperlipidemia, who presented to our hospital with a chief complaint of severe back pain. This was described as sharp, stabbing and with radiation to the substernal area. He was steroid dependent due to his COPD, and was a chronic alcoholic. His home medications included prednisone 10 mg oral (p.o.) daily, fluticasone 230 mcg/salmeterol 21 µg 1 puff daily, montelukast 10 mg p.o. daily, tiotropium 18 mg p.o. daily, simvastatin 40 mg p.o. daily and valsartan 80 mg p.o. twice a day. On presentation, he had stable vital signs with unremarkable physical exam. He had an electrocardiogram which showed normal sinus rhythm. Chest X-ray was unremarkable. On admission, our patient had negative troponins, a sodium level of 135 mmol/l, potassium level of 4.5 mmol/l, creatinine level of 53.4 µmol/l, and a blood sugar level of 134 mmol/l. A computed tomography angiogram was performed which did not show any signs of aortic dissection. An MRI of the back showed that our patient had an acute compression fracture in the 6th thoracic vertebra. 

**Figure 1.**
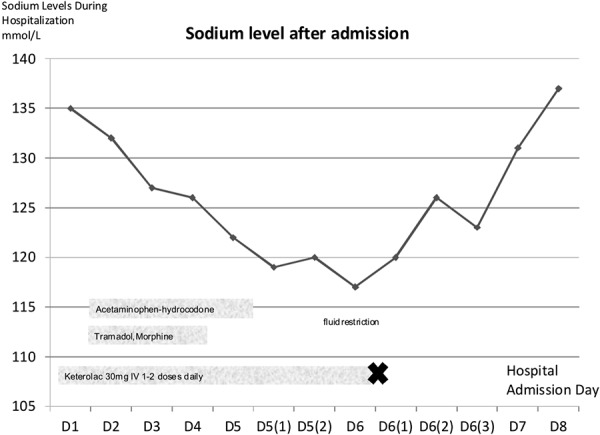
Sodium levels during hospitalization, D = hospital admission day, IV = intravenously. X-day keterolac was stopped.

Our patient was started on ketorolac 30 mg i.v. for pain control and he received 1 – 2 doses of ketorolac per day until Day 6 of admission. Due to poor pain control, tramadol, morphine, and acetaminophen-hydrocodone were added (Figure 1). Our patient’s sodium levels began to drop after admission (Figure 1), and tramadol (discontinued on Day 4), then morphine, acetaminophen-hydrocodone (discontinued on early Day 5) were discontinued. Although our patient was in pain, he denied any nausea and vomiting during this course. On admission Day 5, his sodium level was 122 mmol/l, and he was given i.v. normal saline but this did not improve his sodium levels. His sodium levels continued to decrease to a level of 117 mmol/l on admission Day 6, where he developed altered mental status and patient was transferred to intensive care unit (ICU) for further management. 

On transfer to the medical intensive care unit, he had stable vitals with blood pressure of 108/85 mmHg, and heart rate of 95 beats/minute. Our patient was euvolemic on clinical exam. There were no signs of peripheral edema, nor were there crackles in the lungs. Oral mucosa was moist and he had normal skin turgor. He had a serum osmolality of 267 mmol/kg, urine osmolality of 517 mmol/kg, thyroid-stimulating hormone of 0.57 mIU/l and morning cortisol level of 314.5 nmol/l. He had normal kidney function with a creatine level of 53.04 mmol/l. The urine sodium was 34 mmol/l. These findings were consistent with the diagnosis of SIADH. Our patient was fluid restricted and the keterolac was stopped. He demonstrated improvement of his sodium levels subsequently, maintaining levels of 130 – 140 mmol/l 2 days after stopping keterolac. His mental status also improved and he was discharged from the ICU. 

## Discussion 

We report here a case of SIADH possibly associated with ketorolac. The development of SIADH in our case was multifactorial. The patient’s pain [4], and alcohol withdrawal [5], may have contributed to increased levels of ADH. It is likely that prolonged use of keterolac potentiated the development of SIADH by potentiating the effects of increased ADH. The fact that hyponatremia continued to develop after tramadol, morphine, hydrocodone were discontinued suggests that these medications were not the main cause of hyponatremia. 

Although a rare occurrence, clinicians need to be aware that NSAIDs may be a cause of hyponatremia and SIADH. Studies in humans and animals have shown that the effect of ADH is potentiated by NSAIDs [6]. Prostaglandins are present in the kidney and inhibit the water reabsorption effect of ADH. NSAIDs decrease prostaglandin formation and therefore the effect of ADH is potentiated. However, the development of hyponatremia and SIADH is rare in the setting of NSAID use only. This is because prostaglandins are also present in the central nervous system. In the central nervous system, prostaglandins stimulate the secretion of ADH, therefore the potentiation of the effect of ADH is cancelled out with the use of NSAIDs [7]. Also the initial fall in the plasma sodium concentration also serves as negative feedback that will diminish ADH secretion [8]. 

SIADH in the setting of NSAIDs usually occurs when there are additional factors such as hypovolemia or medications causing nonsuppressable ADH release [8]. Clinically, hyponatremia has been found to develop in neonates when indomethacin was used to treat patent ductus arteriosus. Infants were more likely to develop hyponatremia if they had lower birth weight or were severely ill [9]. Marathon runners are also likely to develop hyponatremia when taking NSAIDs [10], although not all studies showed similar findings [11]. A review of the previously reported case reports found that SIADH and hyponatremia developed when medications such as cyclophosphamide [12] were used, and also in the setting of multiple analgesic use-conjunctional use of NSAIDs with paracetamol or acetylsalicylic acid [13] (similar to our case). Other risk factors in reported cases that may have contributed to the development of SIADH include chronic renal failure, chronic heart failure, and aged older than 65 [3]. Prolonged use (more than 5 days) of keterolac may increase the risk of the development of SIADH. The United States label recommendation calling for a maximum of 5 days of therapy was based on the maximum duration of therapy that had been studied and not due to any data indicating a higher risk with longer duration [14]. However, Strom et al. [15] in a large cohort study found that longer duration of keterolac use was associated increased gastrointestinal bleeding. Although the data is inconclusive, caution is recommended in using keterolac for longer periods of time. 

NSAIDs and SIADH have been reported with ibuprofen [16], indomethacin [17], piroxicam [13], sulindac [18], and diclofenac [19], but no previous cases have been reported for keterolac. As in other NSAIDs, the analgesic effects of keterolac are due to inhibition of prostaglandin synthesis in the periphery. These prostaglandins are released in response to direct trauma in the tissue and mediate pain and inflammation. It is important to note however that there are differences between different NSAIDs [20]. Keterolac has relatively strong analgesic potency and is equivalent to morphine and meperidine. Besides exerting their effect on prostaglandin synthesis peripherally, keterolac may act centrally in the central nervous system and cause the release of endogenous endorphins [21] or have a modulatory effect on opioid receptors [22]. These additional effects may contribute to the development of hyponatremia; for example endorphins have been found to stimulate the production of ADH in animals [23] and also may play an independent role in regulation of water homeostasis [24]. 

In summary, we report a case of SIADH associated with keterolac. Keterolac may have unique properties that predispose to the development of hyponatremia and SIADH. Clinicians need to consider the possibility of NSAIDs in general and keterolac specifically as a cause of hyponatremia and SIADH. Further research regarding risk factors that predispose to the development of hyponatremia in the setting of NSAID use is important to help identify patients who are at high risk of developing this complication. 

## Conflict of interest 

Sian Yik Lim, Ragesh Panikkath, Sharma Prabhakar declare no conflict of interest.
